# Harvesting Stage Regulates the Rheological, Colour and Chemical Properties of *Gracilaria changii* Agar

**DOI:** 10.21315/tlsr2026.37.3.14

**Published:** 2026-10-15

**Authors:** Faten Nur Aisyah Zainuldin, Nurul Elma Sabri, Jalaludin Kassim, Shahidah Md. Nor

**Affiliations:** 1 Department of Technology and Natural Resources, Faculty of Applied Sciences and Technology, Universiti Tun Hussein Onn, UTHM-Campus (Pagoh Branch), Pagoh Education Hub, KM 1, Jalan Panchor, 84600 Muar, Johor, Malaysia;; 2 Department of Agrobiotechnology and Bioscience, Agensi Nuklear Malaysia, 43000 Kajang, Selangor, Malaysia; 3 Laman Alam Jaya Sdn Bhd, Benteng Parit Bulat Laut, Parit Jawa 84150 Muar, Johor, Malaysia

**Keywords:** Gel Syneresis, Agar Yield, Sulphate Content, 3,6-anhydrogalactose, Alkaline Pretreatment, Sineresis Gel, Hasil Agar, Kandungan Sulfat, 3,6-anhidrogalaktosa, Pra-Rawatan Alkali

## Abstract

*Gracilaria changii* is a red seaweed with significant economic potential and has emerged as a promising species for industrial agar production. Despite its commercial importance, limited information is available regarding the influence of harvesting stage on the physicochemical and rheological quality of agar extracted from *G. changii*. Since this seaweed exhibits an isomorphic life cycle that is completed within approximately 30 days–90 days, variations in harvesting stage may significantly affect agar composition and functionality. Thus, identifying the optimal harvesting stage is essential for producing high-quality agar suitable for industrial use. This study evaluated the effect of different harvesting stages; Stage I (30 days–40 days), Stage II (40 days–50 days) and Stage III (60 days and above). Seaweed at different harvesting stages were then subjected to alkaline pretreatment followed by hot-water agar extraction. The extracted agar was evaluated for rheology, colour and chemical properties. This study revealed that stage I was the optimal harvesting stage since the agar had the best rheological properties with high agar yield (7.36%), high transparency (53.39%), low gel syneresis (1.64%) and the highest gel strength (299.94 g/cm^2^) as compared to other stages. Agar extracted from Stages I and II exhibited a more intense red-brown colour than agar obtained from Stage III. In addition, the Stage I agar had a low total sulphate content (4.58%) and a high 3,6-anhydrogalactose content (13.33%), which contribute to low syneresis and excellent gel strength. The finding from this study could be used as standard cultivation practices for both cultivator and marketer to standardise the agar quality that meet the industrial specifications.

## Abstrak

Gracilaria changii ialah sejenis rumpai laut merah yang mempunyai potensi ekonomi tinggi telah muncul sebagai spesies yang berpotensi untuk pengeluaran industri agar. Walaupun kepentingan komersialnya tinggi, maklumat mengenai pengaruh peringkat penuaian terhadap kualiti fizikokimia dan reologi agar yang diekstrak daripada G. changii masih terhad. Memandangkan rumpai laut ini mempunyai kitaran hidup isomorfik lengkap dalam tempoh kira-kira 30–90 hari, variasi peringkat penuaian berkemungkinan memberikan kesan yang ketara terhadap komposisi dan fungsi agar. Oleh itu, penentuan peringkat penuaian optimum adalah penting untuk menghasilkan agar berkualiti tinggi yang sesuai untuk kegunaan industri. Kajian ini menilai kesan peringkat penuaian yang berbeza iaitu Peringkat I (30–40 hari), Peringkat II (40–50 hari) dan Peringkat III (60 hari dan ke atas). Rumpai laut pada peringkat penuaian yang berbeza kemudiannya menjalani prarawatan alkali diikuti dengan pengekstrakan agar menggunakan air panas. Agar yang diekstrak dinilai berdasarkan sifat reologi, warna dan kimia. Kajian ini mendapati bahawa Peringkat I merupakan peringkat penuaian optimum kerana agar yang dihasilkan mempunyai sifat reologi terbaik dengan hasil agar yang tinggi (7.36%), ketelusan tinggi (53.39%), sineresis gel yang rendah (1.64%), dan kekuatan gel tertinggi (299.94 g/cm^2^) berbanding dengan peringkat lain. Agar yang diekstrak daripada Peringkat I dan II menunjukkan warna merah-coklat yang lebih pekat berbanding dengan agar yang diperoleh daripada Peringkat III. Selain itu, agar daripada Peringkat I mempunyai kandungan jumlah sulfat yang rendah (4.58%) dan kandungan 3,6-anhidrogalaktosa yang tinggi (13.33%) menyumbang kepada sineresis yang rendah dan kekuatan gel yang sangat baik. Dapatan kajian ini boleh digunakan sebagai standard amalan penanaman untuk pengusaha dan pengedar untuk menyeragamkan kualiti agar yang dapat memenuhi spesifikasi industri.

**HIGHLIGHTS**

- Harvesting stage significantly modulated the physicochemical and rheological properties of *Gracilaria changii* agar.
- Early harvesting at 30 days–40 days produced the highest agar yield (7.36%) and gel strength (299.94 g cm^−2^)
- Lower sulphate content and higher 3,6-anhydrogalactose levels were associated with reduced syneresis and improved gel performance.

## INTRODUCTION

Agar is composed of several units of D-galactose and 3,6-anhydrogalactose linked together by β-1,3 and α-1,4-glycosidic bonds which can be used as various agents in food production and microbiological media (Lomartire & Gonçalves 2023). Agar can be found in most red algal cell walls, especially *Gracilaria* species (Trongnit *et al*. 2024). *Gracilaria changii* is a well-known red seaweed species that is abundant with agar content. This species belongs to the family Gracilariaceae and found in dark red colour with thallus within 18 cm–22 cm tall and the diameter of the thallus within 1 mm–2 mm (Othman *et al*. 2018). It thrives in various habitats, including estuaries, bays, mangroves, reefs and mudflats (Torres *et al*. 2019). This species is predominant in tropical region particularly in the Southeast Asian countries (Lee *et al*. 2019). *G. changii* has high agar yields with strong gel strength and have good potential for agar production (Lee *et al*. 2019). Therefore, *G. changii* could be considered as an economic importance for agar production.

In industrial applications, quantifying agar yield and quality is crucial, as these attributes directly influence the functioning and market value of agar-based products. Higher agar yield enhances extraction efficiency and manufacturing profitability, thus boosting the economic viability of agar industries (Vuai 2022). High-quality agar is essential in food, pharmaceutical and biotechnology applications in which gel stability, texture and temperature resistance are necessary (Lee *et al*. 2016). For instance, agar with increased gel strength enhances texture retention and thermal stability in bakery fillings, confectionery items and glazes (Khoobbakht *et al*. 2024). Consequently, assessing the determinants influencing agar yield and quality is essential for ensuring sustainable production and reliable industrial performance.

The physical and rheological properties of agar are very important in agar production. First-grade food agar possesses high gel strength within 900 g/cm^2^–1,200 g/cm^2^, gelling temperatures of 32°C–40°C, and melting temperatures of 85°C–95°C (Lim *et al*. 2019). Prior research on *Gracilaria* species has predominantly focused on the effects of environmental factors, including salinity, nutrient availability, temperature and seasonal variations, on agar production and quality (Othman *et al*. 2018; Sade *et al*. 2006). Seasonal and environmental variations may alter seaweed metabolism and biochemical composition due to changes in light intensity, water quality and nutrient dynamics, hence indirectly influencing agar characteristics (Steinhagen *et al*. 2021). However, the effect of harvesting stage represents a different biological factor because it is associated with the physiological maturity and growth duration of the seaweed under cultivation. Distinguishing harvesting stage effects from seasonal or environmental influences is important to identify the optimal cultivation duration that maximises agar quality independent of external environmental fluctuations.

Seaweed harvesting stages are counted from the first day when young thalli was introduced to the cultivation system. In general, most *Gracilaria* species are harvested between 45 days and 60 days of cultivation (Radulovich *et al*. 2015). However, shorter cultivation cycles of approximately 30 days–45 days have been reported in tropical Malaysian cultivation areas such as Semporna, Sabah due to favourable environmental conditions and rapid biomass growth (Sade *et al*. 2020). Harvesting stage is considered an important cultivation parameter because seaweed biochemical composition and structural development continuously change throughout the growth cycle. Previous studies reported that *G. tenuistipitata* achieved the highest biomass yield at approximately 30 days of cultivation, followed by a decline after 40 days (Bokhtiar *et al*. 2022). Nevertheless, most existing studies primarily evaluated biomass production or general seaweed quality, while limited information is available regarding how harvesting stage specifically affects the rheological, colour and chemical characteristics of agar extracted from *G. changii* (Lim *et al*. 2019; Radulovich *et al*. 2015). Moreover, research investigating the effects of harvesting stages in Malaysian culture settings is limited, despite Malaysia being a tropical location conducive to *Gracilaria* cultivation.

This study adopted harvesting stages of 30 days–40 days, 40 days–50 days and over 60 days of cultivation to represent early, intermediate, and extended cultivation periods commonly practised in commercial *Gracilaria* farming systems in Malaysia (Suhaimai *et al*. 2025). Analysing various harvesting stages elucidate the effects of growth duration on agar characteristics regardless of overarching seasonal or environmental impacts. This study aims to assess the impact of various harvesting phases on the rheological qualities, colour features and chemical composition of agar isolated from *G. changii*. The findings of this study provide new insight into the relationship between cultivation duration and agar quality under Malaysian tropical cultivation conditions, which may contribute to the optimisation of harvesting strategies for producing high-quality agar suitable for industrial applications.

## MATERIALS AND METHOD

### Sample Preparation

Fresh *G. changii* was obtained from a seaweed farm at Parit Botak, Muar, Johor, Malaysia (N 1°44’28.5684”, E 103°6’24.4332”). The seaweed was cultivated in a man-made pond using integrated multi-trophic aquaculture (IMTA) cultivation practices with pH maintained between 7.5–8.0 and water temperature within 27°C–32°C. The sample was collected at three different harvesting stages which are Stage I (30 days–40 days), Stage II (40 days–50 days), and Stage III (more than 60 days), with three biological replications representing one maturity level. Each biological replicate was further analysed in triplicate as technical replications, resulting in a total of 27 samples analysed. The harvesting stages were counted from the day of the young thallus introduced to the rope. Upon harvesting, the samples underwent a thorough washing process using filtered sea water to eliminate salt, debris, epiphyte and any foreign matters. The sample was brought back to the laboratory and further washed using tap water (Suhaimai *et al*. 2025). The freshly cleansed samples were then immediately subjected to drying process using oven dryer at 60°C until fully dried. The dried samples were kept in the airtight plastic until further analysis.

### Alkaline Pretreatment

Alkaline pretreatment prior to the extraction was done following the procedure conducted by Yarnpakdee *et al*. (2015). A 50 g of the dried samples were immersed in 500 mL preheated of 5% (w/v) sodium hydroxide (NaOH) solution at 80°C for 30 min using a water bath. After alkaline pretreatment, the removal of the alkali was done by washing the samples with running tap water. The samples were neutralised in 0.025% H_2_SO_4_ with seaweed/solution ratio of 1:50 for 30 min. The seaweed residues were washed repeatedly under tap water until the pH value of the washing water reached 8–8.5.

### Agar Extraction

A 500 mL of distilled water was pre-heated at 90°C using a hot plate. Agar extraction was subsequently performed by immersing the pretreated seaweed samples in the preheated distilled water for 2 h at a seaweed-to-water ratio of 1:150 (w/v). (Martínez-Sanz *et al*. 2019). After that, a filter cloth was used to separate the seaweed and the solution. The solution obtained was poured onto the silicone tray. The solution was cooled at room temperature until it became a gel. The filtrate was frozen overnight at −21°C. Then, the filtrate was thawed at 25°C and dried using oven dryer at 60°C. Finally, agar was ground into powder.

### Rheological Assessment of Agar

#### Determination of yield

The agar extraction yield (dry weight basis) was determined according to Maleki *et al*. (2023). The formula was as follows:


Yield (%)=(Mass of dried agar/mass of dried seaweed)×100

#### Transparency

A potion of 1 mL of agar sample with a concentration of 1% (w/v) was prepared using distilled water. The sample was immediately poured into a cuvette and left for 12 h at 25°C (Zhang *et al*. 2020). The transparency was observed at 700 nm using a spectrophotometer, with distilled water serving as the blank reference.

#### Gel syneresis

The agar with 1.5% (w/v) was pre-heated at 60°C before being poured into the 50 mL of centrifuge tubes. Later, it was cooled at room temperature to form a gel (Khoobbakht *et al*. 2024). The centrifuge tubes were kept at 4°C for 24 h and then centrifuged at 4,200 rpm for 10 min to expel the solvent. Gel syneresis was expressed as a percentage (%) and calculated using the following formula:


$$\text{Gel syneresis }(\%)=\frac{\text{Initial weight}-\text{Final weight}}{\text{Initial weight}}\times 100$$

Where initial weight is the weight of the gel before centrifugation and final weight is the weights of the gel after centrifugation and eliminating the released water.

#### Liquefaction and jellification temperatures

Liquefaction and jellification temperatures were determined according to the procedure conducted by Zhang *et al*. (2020). For the liquefaction temperatures, the sample at a concentration of 1.5% (w/v) was prepared using distilled water and left overnight at 25°C. The liquefaction temperatures were determined by raising the temperature from 50°C to 100°C at a rate of 1°C/min in a water bath. A 5 mm silver bead was put on the surface of the gel, and the liquefaction temperatures were recorded when the bead fell. In parallel, the same concentration of hot agar solution was used to measure the jellification temperature. The test tubes containing the melted agar were tilted up and down until the bead ceased to move. The gelling temperatures were recorded once the agar hardened as indicated by the static glass bead.

#### Gel strength

This procedure was conducted following the method outlined by Lee *et al*. (2019). Texture analyzer (TA-XT2, Stable Micro Systems Ltd., UK) was used to observe the force required to break the agar gel. A 30 mL of 1.5% (w/v) agar gel was prepared in a plastic container with a gel depth of approximately 2 cm–2.5 cm was prepared and allowed to set at 4°C for 12 h. Gel strength was expressed in grams per centimetres.

#### Colour assessment of agar

The colour of the agar was measured using a colorimeter based on CIELAB color space (PCE-CSM Spectrophotometer; PCE Instruments) (Mohibbullah *et al*. 2023). The L* represents the brightness of the sample, ranging from 0 (completely black) to 100 (completely white). The a* measures the colour along the green (−a*) to red (+a*) while the b* measure the colour along the blue (−b*) to yellow (+b*) spectrum. The C* (chroma) representing the colour intensity and h° (hue) describing the actual colour tone, based on the position of the colour in the a*−b* plane was also calculated (Nor *et al*. 2023).

### Determination of Chemical Composition of Agar

#### Total sulphate content

Sulphate content was measured using BaCl_2_-gelatin method (Martínez-Sanz *et al*. 2019). The total of 1 mL of 1 M HCl was used to hydrolyse 5 mg of agar powder at the boiling point of 100°C for 5 h. After 5 h, the sample was allowed to cool at 25°C. Filter paper was used to filter the solution. The total of 3.8 mL of 3% trichloroacetic acid and 1 mL of BaCl_2_-gelatin reagent was transferred to test tube. 0.2 mL of the sample solution was put into the the test tube and incubate at 25°C for 15 min. The absorbance was read at 360 nm. Distilled water was used as a blank. K_2_SO_4_ was used to build a standard curve. The sulphate content was then calculated and expressed as a percentage on a dry weight basis.

#### 3,6-anhydrogalactose content

A colourimetric assay was used to determine the content of 3,6-anhydrogalactose (3,6-AG) in the agar (Yarnpakdee *et al*. 2015; Yaphe & Arsenault 1965). Cold-resorcinol acetal reagent was done by preparing 0.907 Mm resorcinol and 2.06 μM acetal in a dark screw cap bottle. The total of 2 mL agar (0.04 mg/mL) was put in a test tube that contain 10 mL of resorcinol-acetal reagent. The tube was put in an ice bath for 15 min, soaked in 20°C water bath for 4 min and then incubated at 80°C for 10 min. The absorbance was read at 555 nm. D-fructose was used as a standard. The 3,6-anhydrogalactose content was calculated and expressed as percentage (% w/w of 3,6-anhydrogalactose in agar) (Lee *et al*. 2019).

### Statistical Analysis

Statistical analysis was performed using IBM SPSS Statistics (IBM Corp., Armonk, NY, USA) following the method described by Rahim *et al*. (2022). Prior to ANOVA analysis, data normality was evaluated using Q–Q (quantile–quantile) plots, where the data were considered approximately normally distributed when the plotted points closely followed the diagonal reference line without substantial deviation. The effects of different harvesting stages were analysed using one-way analysis of variance (ANOVA), and mean separation was performed using Tukey’s post hoc test at *p* < 0.05.

## RESULTS AND DISCUSSION

### Rheological Properties of Agar

#### Agar yield at different harvesting stages

The yield of agar extracted from different harvesting stages is shown in Table 1. The agar yield obtained from this was in the range of 5.39%–7.36%, which is lower than the value of agar in *G. changii* at 18.08%–24.3% (Lee *et al*. 2019; Lim *et al*. 2019). The lower agar yield obtained in this study may be attributed to differences in the extraction protocol, where conventional hot-water extraction combined with sodium hydroxide alkaline pretreatment was applied. Alkaline pretreatment, particularly under prolonged heating conditions, may partially hydrolyse agar polysaccharides and reduce their molecular integrity, leading to lower agar recovery (Li *et al*. 2021). In addition, the freeze–thaw dehydration process used during agar recovery could have promoted structural disruption and loss of soluble agar fractions through water separation during thawing (Charoenrein & Owcharoen 2016). The repeated washing and filtration steps may have further reduced agar yield through the loss of fine polysaccharide particles and partially dissolved agar molecules (Wang *et al*. 2017). Compared to previous conventional alkaline extraction studies, the current study employed a simple laboratory-scale hot-water extraction technique supplemented with sodium hydroxide pretreatment and freeze-thaw dehydration. Previous study demonstrated that a high agar yields and gel strengths can be achieved through optimised alkaline extraction parameters, which included a regulated alkali concentration, pressure-assisted heating, elevated extraction temperatures and enhanced filtration efficacy, which were not applied in the present study (Martínez-Sanz *et al*. 2021; Xio *et al*. 2021; Al-Nahdi *et al*. 2015).

Notably, stage I delivered maximum agar production at 7.36%, in contrast to stage II and III, which yielded 5.78% and 5.39%, respectively. In addition, Stage I generated the maximum agar yield at 7.36%, whereas Stage II and Stage III gave only 5.78% and 5.39%, respectively. The increased agar yield observed at the initial harvesting stage may be linked to the vigorous vegetative growth phase of younger *G. changii* thalli, wherein the cell wall polysaccharides retain their structural integrity and are more easily extractable (Suhaimai *et al*. 2025). During the beginning of growth phase, agar polysaccharides exhibited reduced sulfation levels and increased amounts of structurally stable galactans, facilitating agar recovery (Chumsook *et al*. 2023). In contrast, prolonged cultivation may promote physiological ageing and progressive structural modification of cell wall components, including increased sulfation and partial degradation of polysaccharide networks, which could reduce extraction efficiency and agar yield (Chumsook *et al*. 2023).

Transparency is one of the characteristics that can influence consumer choice because it determines the compatibility of the agar to be used in certain products (Xiao *et al*. 2019). Agar is widely used as a multipurpose agent in the food sector; therefore, higher transparency is required to ensure that the addition of agar in food products for gelling purposes does not affect their appearance and colour. In the present study, the highest transparency was observed in agar obtained from Stage I at 53.39%, as compared to Stages II and III which have 51.24% and 49.63%, respectively. The transparency of food-grade agar was reported at 56.8% while 64.4% and 65.6% for bleached agar and biochemical agar, respectively (An *et al*. 2021). Nevertheless, the qualification of transparency of high-quality agar has not been well described. Specific values should be determined to ensure consistency and quality for industrial and consumer applications. Transparent agar has been shown to improve the appearance and functionality of food products, making it a preferred choice for high-quality culinary applications (An *et al*. 2021).

Syneresis is the key indicator of agar stability and quality during long term storage (Khoobbakht *et al*. 2024). The syneresis phenomenon could be seen when the water starts to separate from the agar due to the dysfunction in gel network which result in the texture and appearance degradation (Khoobbakht *et al*. 2024). In the previous study, *G. changii* and *G. edulis* collected from the rocky shore showed the highest syneresis at 13.09% and 13.64%, respectively compared to the samples collected from the mangrove swamp (Lee *et al*. 2016). In this study, this species was cultivated using IMTA system in the man-made pond where the gel syneresis was in range of 1.64%–1.87%. These values showed that man-grown seaweed has better gel syneresis compared to natural *G. changii*. Nevertheless, to the author’s best knowledge, no established standard for acceptable agar syneresis value has been well reported. Thus, further study on agar syneresis should be conducted to ensure that the values could be standardised in the market. In this present study, the lowest gel syneresis was obtained at Stage I (1.64%). This indicates that agar extracted from Stage I exhibited relatively good gel stability, as reflected by its low syneresis value. The syneresis obtained in this study was within the range commonly reported for food-grade agar gels (approximately 0.5%–2.5%), suggesting acceptable water-holding capacity and gel network stability under the present extraction conditions (Lin *et al*. 2023).

Different agency bodies specify different jellification temperature of agar when rating the agar quality. For instances, the jellification temperature at a range of 32°C–40°C is required to be graded as first-grade food agar as specified by Japanese Specifications for Processed Agar (JPSA) (Lim *et al*. 2019). Meanwhile, the US Pharmacopoeia (USP) specify the agars must have a jellification temperature ranging from 32°C–39°C as a premium agar (Xiao *et al*. 2019). In this study, the jellification temperatures obtained from all stages were in the range of 35.8°C–37.1°C, which fit the jellification temperature required by both USP and JPSA. Evaluating the harvesting stage, stage III has the lowest jellification temperature (35.8°C) in comparison with Stage I and II. For liquefaction temperatures, the agar should have liquefaction temperatures in range of 85°C–95°C to be graded as high-quality agar specified by both JPSA and USP (Lee *et al*. 2016; Lim *et al*. 2019). In this study, the liquefaction temperatures of agar from all stages met the requirement of both JPSA and USP standards for agar as the temperatures were in the range of 87.4°C–89.7°C (Xio *et al*. 2020).

Gel strength is often used as a reference parameter for determining the agar quality. First-grade food agar is required to have gel strength at a range of 900 g/cm^2^–1,200 g/cm^2^ as specified by JPSA (Lim *et al*. 2019). In microbiological applications, high-quality agar with gel strengths of more than 700 g/cm^2^ is often required on the global market, while the gel strength values (30 g/cm^2^–200 g/cm^2^) could be preferred for food industry applications (Mohibbullah *et al*. 2023). For second-grade agar, the gel strength must be in the range of 300 g/cm^2^–500 g/cm^2^ (Freile-Pelegrín & Murano 2005). The gel strength obtained from this study is in the range of 132.21 g/cm^2^–299.94 g/cm^2^, approaching the second-grade agar. The relatively low gel strength obtained in this study suggests that the current extraction protocol was insufficient to produce premium-grade agar. This may be associated with incomplete conversion of L-galactose-6-sulphate into 3,6-anhydrogalactose during alkaline pretreatment, resulting in higher sulphate retention and weaker gel network formation (Chumsook *et al*. 2023). Based on the gel strength classification, the agar extracted in this study may be more suitable for selected food applications requiring moderate gel rigidity, such as desserts, stabilisers and thickening agents, rather than microbiological or bacteriological agar applications that generally require gel strengths exceeding 700 g/cm^2^ (Khoobbakht *et al*. 2024). Furthermore, the agar still demonstrated acceptable jellification and liquefaction temperatures, indicating its potential functionality in low-to-intermediate grade food formulations. The gel strength obtained from *G. changii* was consistent with other *G. changii* (132.78 g/cm^2^–201.33 g/cm^2^) species that inhabited diverse environmental conditions (Lee *et al*. 2016). Overall, Stage I demonstrated the most favourable physical characteristics of agar, including the highest agar yield, transparency and gel strength, together with the lowest gel syneresis compared to Stage II and Stage III. These findings suggest that Stage I may represent the optimal harvesting stage for producing agar with improved physical and rheological properties under the present extraction conditions. Nevertheless, further optimisation of extraction and purification protocols is still necessary before considering broader industrial applications

#### Colour analysis of agar extracted at different harvesting stages

Colour is an important quality attribute of agar because it directly influences sensory acceptance and consumer preference, particularly in food and commercial applications (Mohibbullah *et al*. 2023). The colour attributes of agar obtained from various harvesting phases are illustrated in Fig. 1 and Table 2. Significant variations in colour characteristics were observed across the harvesting stages, suggesting that cultivation duration affected pigment retention and the visual appearance of the extracted agar (Suhaimai *et al*. 2025). The lightness (L*) values first reduced from Stage I to Stage II before increasing significantly (*p* < 0.05) at Stage III, indicating that the agar became substantially lighter during extended harvesting stages. All agar samples exhibited positive a* and b* values, indicating that the extracted agar retained a reddish-yellow tone. The gradual decline in a* values from Stage I to Stage III indicated that extended harvesting diminished the red-brown intensity of the agar. In turn, the chroma (c*) values diminished markedly during Stage III, indicating less colour saturation and a more reduced appearance relative to Stages I and II harvesting phases. The hue angle readings of 31.8*°*–41.27° further confirmed that the agar colour exhibited a spectrum between reddish and yellowish tones (Fig. 1).

The pronounced red-brown colour found in agar derived from Stage I may correlate with the retention of residual photosynthetic pigments and phenolic chemicals in younger and metabolically active thalli (Suhaimai *et al*. 2025). Conversely, extended development during Stage III may have facilitated physiological ageing, pigment breakdown and oxidative alteration of cellular constituents, leading to the development of a lighter yellowish agar. Prolonged cultivation may induce structural degradation of seaweed tissues, potentially affecting the stability of pigment-associated chemicals inside the cell wall matrix (Chumsook *et al*. 2023). Among the three harvesting stages, Stage I demonstrated the most favourable colour characteristics with higher colour intensity and better visual appearance, suggesting improved pigment retention and reduced degradation during earlier cultivation stages. The findings suggest that earlier harvesting phases may be more appropriate for producing agar with superior colour quality and appearance stability under the current extraction circumstances.

#### Total sulphate content and 3,6-anhydrogalactose content in agar extracted at different harvesting stages

The total sulphate content and 3,6-anhydrogalactose (3,6-AG) are significant determinants that profoundly impact the rheological properties of agar. The current investigation revealed that agar harvested during Stage I had the lowest sulphate content (4.58%) and the greatest 3,6-AG content (13.33%), while extended harvesting stages demonstrated an increase in sulphate accumulation and a decrease in 3,6-AG content (Table 3). Similar sulphate concentrations have been previously documented in *Gracilaria* species gathered from various environments and extraction methodologies (Yarnpakdee *et al*. 2015; Li *et al*. 2021). The observed increase in sulphate concentration from Stage I to Stage III may signify increased accumulation of sulphated galactans throughout the maturation and physiological ageing of the thalli (Sasuga *et al*. 2017). Sulphate groups impart negatively charged substituents to the agar polymer chains, weakening intermolecular hydrogen bonding and disrupting the formation of stable double-helix structures during gelation (Xio *et al*. 2019). As a result, excessive sulphation compromises the three-dimensional gel network, leading to diminished gel strength and increased gel syneresis (Chen *et al*. 2020). This trend was distinctly observed in Stage III agar, which possessed the highest sulphate concentration and thus causing a decreased gel strength and high syneresis values (Table 1). In contrast, the improved agar quality observed in Stage I could potentially be linked to younger, metabolically active thalli that possess structurally intact polysaccharides with reduced sulfation and increased efficiency in 3,6-AG production. Thus, it can be concluded that early harvesting phases are more suitable for creating agar with improved gel properties and rheological stability.

## CONCLUSIONS

This study revealed that different harvesting phases had a substantial impact on the quality of *G. changii* agar. Stage I produced agar with the most desirable quality characteristics, including higher agar yield, improved gel strength, lower syneresis, good colour retention, lower sulphate content and higher 3,6-anhydrogalactose (3,6-AG) content. These findings indicate that earlier harvesting stages are more capable of producing agar with better physical and chemical properties under present extraction conditions. Overall, this work provides information on the optimal of harvesting procedures for *G. changii* cultivation. Further research should focus on optimising extraction and purification processes to improve agar recovery and gel quality for broader industrial applications.

## Figures and Tables

**FIGURE 1:**
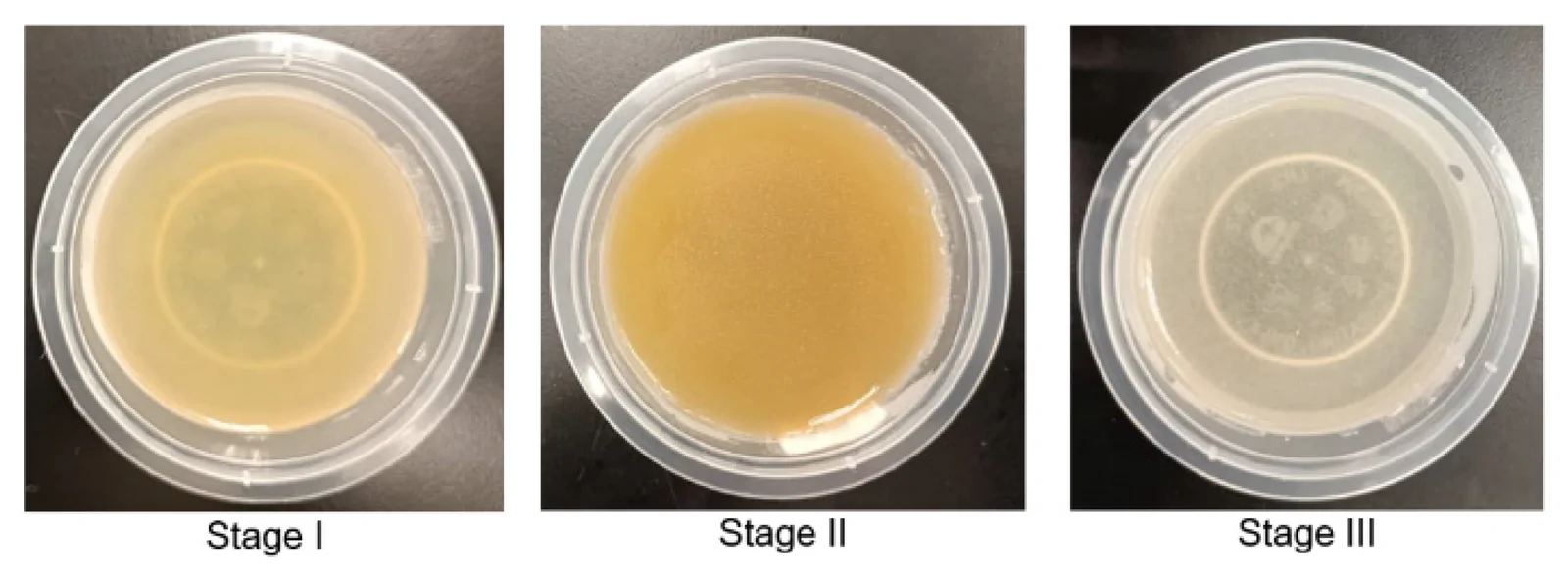
Agar extracted at different harvesting stages.

**TABLE 1:** Physical characteristics of agar extracted from *G. changii* at different harvesting stages.

Agar	Yield (%)	Transparency (%)	Gel syneresis (%)	Jellification temperatures (°C)	Liquefaction temperatures (°C)	Gel strength (g/cm^2^)
Stage I	7.36 ± 0.18^a^	53.39 ± 0.04^a^	1.64 ± 0.03^b^	37.1 ± 0.44^a^	87.5 ± 0.10^b^	299.94^a^
Stage II	5.78 ± 0.33^b^	51.24 ± 0.01^b^	1.87 ± 0.07^a^	36.5 ± 0.20^a^	87.4 ± 0.23^b^	239.85^b^
Stage III	5.39 ± 0.26^c^	49.63 ± 0.01^c^	1.84 ± 0.04^a^	35.8 ± 0.15^b^	89.7 ± 0.29^a^	132.21^c^

*Notes*: Result was expressed in means ± standard deviation (*n* = 27) followed by the same letter in the same column was not significantly different at (*p* > 0.05) according to Tukey’s post-hoc tests.

**TABLE 2:** The effects of different harvesting stages of *G. changii* on agar colour.

Agar	L*	a*	b*	c*	h°
Stage I	65.5 ± 0.95^ab^	3.3 ± 0.1^a^	22.87 ± 0.67^b^	23.09 ± 0.63^b^	36.53 ± 0.46^b^
Stage II	62.9 ± 1.56^b^	2.37 ± 0.8^ab^	35.87 ± 2.02^a^	35.94 ± 2.09^a^	41.27 ± 0.58^a^
Stage III	66.4 ± 0.85^a^	1.77 ± 0.46^b^	7.63 ± 0.15^c^	7.85 ± 0.24^c^	31.8 ± 1.56^c^

*Note*: Result was expressed in means ± standard deviation (*n* = 27) followed by the same letter in the same column was not significantly different at (*p* > 0.05) according to Tukey’s post-hoc tests.

**TABLE 3:** Total sulfate content and 3,6-anhydrogalactose content in agar at different maturation stages.

Agar	Total sulfate content, % (w/w)	3,6-anhydrogalactose content, % (w/w)
Stage I	4.58 ± 0.08^c^	13.33 ± 0.22^a^
Stage II	5.61 ± 0.02^b^	10.19 ± 0.08^b^
Stage III	6.88 ± 0.03^a^	9.73 ± 0.54^c^

*Note*: Result was expressed in means ± standard deviation (*n* = 27) followed by the same letter in the same column was not significantly different at (*p* > 0.05) according to Tukey’s post-hoc tests.

## References

[b1-tlsr-37-3-307] Al-Nahdi ZM, Al-Alawi A, Al-Marhobi I, Al-Zefiti A (2015). Optimization of yield and chemical properties of agar extracted from *Melanothamnus somalensis* from Oman Sea. Journal of Environmental Science and Engineering B.

[b2-tlsr-37-3-307] An D, Xiao Q, Zhang C, Cai M, Zhang Y, Weng H, Chen F, Xiao A (2021). Preparation, characterization, and application of high-whiteness agar bleached with hydrogen peroxide. Food Hydrocolloids.

[b3-tlsr-37-3-307] Bokhtiar SM, Ali MA, Chowdhury MAZ, Ahmed KU, Hassan MK, Ahmed M, Bhuiyan MS, Mashuk OF, Rahman MM, Salam MA, Rafiquzzaman SM, Hossain MF (2022). Yield improvement of *Gracilaria tenuistipitata* by optimizing different aspects in coast of Cox’s Bazar, Bangladesh. Scientific Reports.

[b4-tlsr-37-3-307] Chen H, Xiao Q, Weng H, Zhang Y, Yang Q, Xiao A (2020). Extraction of sulfated agar from *Gracilaria lemaneiformis* using hydrogen peroxide-assisted enzymatic method. Carbohydrate Polymers.

[b5-tlsr-37-3-307] Charoenrein S, Owcharoen K (2016). Effect of freezing rates and freeze-thaw cycles on the texture, microstructure and pectic substances of mango. International Food Research Journal.

[b6-tlsr-37-3-307] Chumsook K, Praiboon J, Fu X (2023). Sulfated galactans from agarophytes: Review of extraction methods, structural features, and biological activities. Biomolecules.

[b7-tlsr-37-3-307] Freile-Pelegrín Y, Murano E (2005). Agars from three species of *Gracilaria* (Rhodophyta) from Yucatán Peninsula. Bioresource Technology.

[b8-tlsr-37-3-307] Khoobbakht F, Khorshidin S, Bahmanyar F, Hosseini SM, Aminikhah N, Farhoodi M, Mirmoghtadaie L (2024). Modification of mechanical, rheological and structural properties of agar hydrogel using xanthan and locust bean gum. Food Hydrocolloids.

[b9-tlsr-37-3-307] Lee WK, Lim PE, Phang SM, Namasivayam P, Ho CL (2016). Agar properties of *Gracilaria* species (Gracilariaceae, Rhodophyta) collected from different natural habitats in Malaysia. Regional Studies in Marine Science.

[b10-tlsr-37-3-307] Lee WK, Lim YY, Ho CL (2019). pH affects growth, physiology and agar properties of agarophyte *Gracilaria changii* (Rhodophyta) under low light intensity from Morib, Malaysia. Regional Studies in Marine Science.

[b11-tlsr-37-3-307] Li Y, Zhao M, Gomez LP, Senthamaraikannan R, Padamati RB, O’Donnell CP, Tiwari BK (2021). Investigation of enzyme-assisted methods combined with ultrasonication under a controlled alkali pretreatment for agar extraction from *Gelidium sesquipedale*. Food Hydrocolloids.

[b12-tlsr-37-3-307] Lim YY, Lee WK, Lim PE, Phang SM, Leow ATC, Namasivayam P, Abdullah JO, Ho CL (2019). Expression analysis of potential transcript and protein markers that are related to agar yield and gel strength in *Gracilaria changii* (Rhodophyta). Algal Research.

[b13-tlsr-37-3-307] Lin HTV, Tsai JS, Liao HH, Sung WC (2023). The effect of hydrocolloids on penetration tests and syneresis of binary gum gels and modified corn starch–gum gels. Gels.

[b14-tlsr-37-3-307] Lomartire S, Gonçalves AMM (2023). Algal phycocolloids: Bioactivities and pharmaceutical applications. Marine Drugs.

[b15-tlsr-37-3-307] Maleki N, Roomiani L, Tadayoni M (2023). Microwave-assisted extraction optimization, antimicrobial and antioxidant properties of carrageenan from red algae (*Gracilaria acerosa*). Journal of Food Measurement and Characterization.

[b16-tlsr-37-3-307] Martínez-Sanz M, Gomez-Barrio LP, Zhao M, Tiwari B, Knutsen SH, Ballance S, Zobel HK, Nilsson AE, Krewer C, Ostergren K, Lopez-Rubio A (2021). Alternative protocols for the production of more sustainable agar-based extracts from *Gelidium sesquipedale*. Algal Research.

[b17-tlsr-37-3-307] Martínez-Sanz M, Gómez-Mascaraque LG, Ballester AR, Martínez-Abad A, Brodkorb A, López-Rubio A (2019). Production of unpurified agar-based extracts from red seaweed *Gelidium sesquipedale* by means of simplified extraction protocols. Algal Research.

[b18-tlsr-37-3-307] Mohibbullah M, Talha MA, Baten MA, Newaz AW, Choi JS (2023). Yield optimization, physicochemical characterizations, and antioxidant properties of food grade agar from *Gracilaria tenuistipitata* of Cox’s Bazar coast, Bangladesh. Food Science & Nutrition.

[b19-tlsr-37-3-307] Nor SM, Ding P, Chun TJ (2023). Locule position and thawing duration affect postharvest quality of freshly cryo-frozen Musang King Durian fruit. Pertanika Journal of Tropical Agricultural Science.

[b20-tlsr-37-3-307] Othman MNA, Hassan R, Harith MN, Md Sah ASR (2018). Morphological characteristics and habitats of red seaweed *Gracilaria* spp. (Gracilariaceae, Rhodophyta) in Santubong and Asajaya, Sarawak, Malaysia. Tropical Life Sciences Research.

[b21-tlsr-37-3-307] Radulovich R, Neori A, Valderrama N, Reddy CRK, Reddy HC, John F, Tiwari BK, Troy DJ (2015). Farming of seaweeds. Seaweed sustainability food and non-food applications.

[b22-tlsr-37-3-307] Rahim NFA, Muhammad N, Abdullah N (2022). Optimum extraction condition of *Anacardium occidentale* on antiurolithiatic activities (in-vitro). Sains Malaysiana.

[b23-tlsr-37-3-307] Sade A, Ali I, Ariff MRM (2006). The seaweed industry in Sabah, East Malaysia. Jati: Journal of Southeast Asian Studies.

[b24-tlsr-37-3-307] Sasuga K, Yamanashi T, Nakayama S, Ono S, Mikami K (2017). Optimization of yield and quality of agar polysaccharide isolated from the marine red macroalga *Pyropia yezoensis*. Algal Research.

[b25-tlsr-37-3-307] Suhaimai NN, Zainuldin FNA, Kassim J, Nor SM (2025). Morphology and nutritional composition of *Gracilaria changii* at different maturation stages. Pertanika Journal of Tropical Agricultural Science.

[b26-tlsr-37-3-307] Steinhagen S, Enge S, Cervin G, Larsson K, Edlund U, Schmidt AEM, Wahistrom N, Kollander B, Pavia H, Undeland I, Toth GB (2022). Harvest time can affect the optimal yield and quality of Sea lettuce (*Ulva fenestrata*) in a sustainable sea-based cultivation. Frontiers in Marine Science.

[b27-tlsr-37-3-307] Torres MD, Flórez-Fernández N, Domínguez H (2019). Integral utilization of red seaweed for bioactive production. Marine Drugs.

[b28-tlsr-37-3-307] Trongnit J, Mayakun J, Kaewtatip K (2024). The effect of agar from the seaweed *Gracilaria fisheri* on properties of biodegradable starch foam. International Journal of Biological Macromolecules.

[b29-tlsr-37-3-307] Vuai SAH (2022). Characterization of agar extracted from *Gracilaria* species collected along Tanzanian Coast. Heliyon.

[b30-tlsr-37-3-307] Wang L, Shen Z, Mu H, Lin Y, Zhang J, Jiang X (2017). Impact of alkali pretreatment on yield, physico-chemical and gelling properties of high quality agar from *Gracilaria tenuistipitata*. Food Hydrocolloids.

[b31-tlsr-37-3-307] Xiao Q, Weng H, Ni H, Hong Q, Lin K, Xiao A (2019). Physicochemical and gel properties of agar extracted by enzyme and enzyme-assisted methods. Food Hydrocolloids.

[b32-tlsr-37-3-307] Yaphe W, Arsenault GP (1965). Improved resorcinol reagent for the determination of fructose, and of 3,6-anhydrogalactose in polysaccharides. Analytical Biochemistry.

[b33-tlsr-37-3-307] Yarnpakdee S, Benjakul S, Kingwascharapong P (2015). Physico-chemical and gel properties of agar from *Gracilaria tenuistipitata* from the lake of Songkhla, Thailand. Food Hydrocolloids.

[b34-tlsr-37-3-307] Zhang C, An D, Xiao Q, Weng H, Zhang Y, Yang Q, Xiao A (2020). Preparation, characterization, and modification mechanism of agar treated with hydrogen peroxide at different temperatures. Food Hydrocolloids.

